# Prognostic Value of Tumor Regression Grade After Chemotherapy Versus Chemoradiotherapy in Patients Undergoing Neoadjuvant Treatment for Locally Advanced Esophageal Adenocarcinoma

**DOI:** 10.1245/s10434-025-17264-2

**Published:** 2025-05-05

**Authors:** Giovanni Capovilla, Elisa Sefora Pierobon, Lucia Moletta, Alessia Scarton, Maria Elisa Sciuto, Evangelos Tagkalos, Eren Uzun, Carlo Alberto De Pasqual, Cecilia Turolo, Gianpietro Zanchettin, Federica Riccio, Luca Provenzano, Renato Salvador, Felix Berlth, Jacopo Weindelmayer, Sara Lonardi, Sara Galuppo, Simone Giacopuzzi, Giovanni De Manzoni, Peter Grimminger, Michele Valmasoni

**Affiliations:** 1 Department of Surgery, Oncology and Gastroenterology (DISCOG), University of Padova, Padua, Italy; 2 Unit of Chirurgia Generale 1, University Hospital of Padova, Padua, Italy; 3 Department of General, Visceral and Transplant Surgery, University Medical Center of the Johannes Gutenberg University, Mainz, Germany; 4 Department of General and Upper G.I. Surgery, University of Verona, Verona, Italy; 5 Klinik für Allgemein-, Viszeral- und Transplantationschirurgie, Uniklinik Tübingen, Tübingen, Germany; 6 Medical Oncology, Istituto Oncologico Veneto, Padua, Italy; 7 Radiotherapy, Istituto Oncologico Veneto, Padua, Italy

## Abstract

**Background:**

The higher rate of pathological complete response (pCR) after neoadjuvant chemoradiotherapy (NACRT) is an argument to support this treatment. However, previous studies have not demonstrated a survival benefit of NACRT for adenocarcinoma (ADC) compared with neoadjuvant chemotherapy (NACT) and the correlation between pathological tumor response (pTR) and survival is unclear. We aimed to verify whether the prognostic value of pTRis influenced by the type of neoadjuvant treatment performed.

**Methods:**

Patients with ADC who underwent NACT or NACRT and surgery between 2015 and 2020 were included. The correlation between pTR and overall survival (OS) and disease-free survival (DFS) after both treatments was evaluated by using Kaplan-Meier analysis. pTR was assessed by using the Mandard tumor regression grade (TRG).

**Results:**

Overall, 563 patients were included; 278 received NACT, and 285 NACRT. The incidence of pCR was significantly higher after NACRT (24.6% vs. 11.2%, *p* < 0.0001). The TRG of both node-negative (pN0) and node-positive (pN+) patients significantly correlated with the 5 years OS after NACT (pN0 *p* = 0.03, pN+ *p* = 0.01). The same result was not detected in NACRT patients (pN0 *p* = 0.98, pN+ *p* = 0.23). The 5-year DFS of the patients with pCR was higher in the NACT group (84% vs. 66.5%, *p* = 0.05). The proportion of patients showing distant recurrences was significantly higher in the NACRT group (35.4% vs. 23.8%, *p* = 0.009).

**Conclusions:**

Tumor regression grade was significantly associated with survival after NACT, but not with NACRT. Despite a lower rate of pCR, both OS and, especially, DFS of patients with pCR improved after NACT compared with NACRT.

**Supplementary Information:**

The online version contains supplementary material available at 10.1245/s10434-025-17264-2.

Multimodal treatment is nowadays the standard of care for locally advanced esophageal adenocarcinoma. However, it is unclear which is the best approach, being both perioperative chemotherapy (NACT) and neoadjuvant chemoradiotherapy (NACRT) viable options.^1,2^

The recently published ESOPEC randomized controlled trial (RCT) currently represents the most relevant study on this topic and showed an improved survival after perioperative chemotherapy with FLOT compared with preoperative chemoradiotherapy, among patients with resectable esophageal adenocarcinoma.^3^ Previously published RCTs concluded that the long-term prognosis is comparable between the two treatment arms, despite showing a higher rate of radical resection, pathological complete response (pCR) and a higher tumor regression grade (TRG) in the NACRT arm.^4^ Several retrospective studies analyzed the different outcomes of the two treatment modalities, showing similar results.^5–9^

One of the more controversial points is how to combine the survival outcomes with the well-known relationship between pathological tumor response and long-term results.^10^ It seems that the prognosis of patients depends not only on the pathological stage after a multimodal treatment, but also on the type of treatment. Recently published data showed a significantly improved disease-free survival (DFS) after NACT compared with NACRT in patients with pCR. This was mainly correlated with a higher incidence of distant recurrence in the NACRT group.^11^

These data appear to question the effectiveness of chemotherapy incorporated in NACRT regimens.^12^ In this context, tumor regression grade may not be considered as a prognostic factor *per se* but must be correlated with preoperative treatment.^13^

The purpose of the study is to verify whether the prognosis of patients with esophageal adenocarcinoma after radical surgery, stratified according to TRG, is influenced by the type of neoadjuvant treatment performed. Our study hypothesis was that while tumor regression grade significantly correlates with survival after NACT, there might be no correlation after NACRT. Despite a higher rate of complete pathological response after NACRT, there might be no difference in survival between the two treatments, and complete responders might show better survival after NACT. Secondarily, we sought to evaluate whether patients showing a pCR and a “near complete” response show comparable survival outcomes.

## Materials and Methods

### Ethical Considerations

The study was conducted in accordance with the Declaration of Helsinki. The study protocol was approved by the Institutional Review Board of the Department of Surgery, Oncology, and Gastroenterology of Padova University Hospital. Ethical approval for this study (Ethical Committee N. 2175CESC) was provided by the Ethical Committee of Verona University Hospital, Verona, Italy, May 15, 2019.

### Study Design

In this international multicentric retrospective cohort study, all consecutive patients treated for esophageal adenocarcinoma (ADC) between January 2015 and December 2020, with follow-up of patients until December 2023 were recruited. All institutions were referral centers for the treatment of esophageal cancer with a case volume of at least 70 esophagectomies per year.

The surgical radicality and postoperative morbidity and mortality were compared between NACT and NACRT patients. Differences in terms of overall survival (OS) and DFS between the two groups were evaluated. A further subset analysis was performed to evaluate the correlation between tumor response as measured by tumor regression grade and survival within each treatment group.

Univariate and multivariate analyses were performed to evaluate clinical factors correlating with survival.

### Study Population

Patients meeting the following inclusion criteria were enrolled: (1) subjects aged ≥18 years old at the time of primary treatment; (2) histologically confirmed ADC of the esophagus or esophagogastric junction (EGJ) (Siewert 1 and 2) treated with NACT or NACRT and surgery; (3) at least 3 years of follow-up after primary treatment.

Exclusion criteria were (1) patients with squamous cell carcinoma; (2) T4b or M+ cancer at presentation; (3) patients who underwent definitive chemoradiotherapy; (4) patients with cervical or Siewert 3 cancer; (5) patients who underwent palliative surgical procedures; (6) patients with radiation-induced cancers; (7) patients with other synchronous malignancies; (8) patients with R1 or R2 resections; (9) patients with a nonadequate lymphadenectomy (<12 lymph nodes); (10) patients who did not complete the scheduled preoperative treatment (NACT or NACRT).

### Collected Data and Definitions

Patients’ preoperative demographic and clinical data included age, sex, weight, body mass index (BMI). The general preoperative condition of the patients and the operative risk were evaluated by using the American Society of Anesthesiology (ASA) classification and the Charlson’s Comorbidity Index (CCI).^14,15^

The recorded tumor characteristics included tumor location (upper, middle, or distal esophagus and EGJ). The clinical and pathological stage of ADC was classified according to the 8th edition of the AJCC classification.^16^

The pathological response of the tumor to NACT or NACRT was quantified according to Mandard’s TRG.^17^ The TRG was evaluated prospectively on every specimen as part of the standard daily clinical practice at each participating institution. The pathology reports of the patients enrolled in this study were subsequently reviewed retrospectively by two expert pathologists at each center to confirm the TRG scores.

The following definitions were applied to NACT and NACRT:

- NACT: systemic perioperative regimens, including at least two drugs
- NACRT: preoperative regimens including at least 2 drugs and at least 41.4 Gy of radiation

The planning, scheduling, and management of the neoadjuvant treatment was based on multidisciplinary discussion at the local tumor boards of the participating centers.

The following surgical procedures were included in the study: left thoraco-abdominal esophagectomy; Ivor-Lewis or Mc Keown esophagectomy. Open, fully minimally invasive (including robotic assisted) or hybrid approaches (laparoscopic abdomen and open thorax or vice versa) were included. Only radical surgical procedures that included at least a standard two-field lymphadenectomy were considered recruitable. Trans-hiatal operations were excluded.

Postoperative 90-day morbidity was classified according to the Esophagectomy Complications Consensus Group ECCG and graded according to the Clavien-Dindo classification.^18,19^ Postoperative mortality was defined as deaths from all causes occurring within 90 days of the operation.

Disease-free survival was defined as the time from the surgical procedure to disease recurrence or death, regardless of the cause. The diagnosis of disease recurrence during follow-up required confirmation of pathological PET-CT uptake of newly identified lesions on follow-up CT scan and/or histological confirmation of lesions amenable to biopsy.

Locoregional recurrences were defined as those that occur in the anastomotic or perianastomotic region of the gastric conduit or the esophageal remnant and those that involve the lymph nodes included in the two-field lymphadenectomy. Distant recurrences were defined as the presence of disease in the outfield lymph nodes (cervical, supraclavicular, or below the level of the pancreas), peritoneal carcinomatosis, malignant pleural effusions, or metastases to distant organs. Overall survival was defined as the time from the surgical procedure to death, regardless of the cause.

### Statistical Analysis

Statistical analysis was conducted by a biostatistician. Continuous variables were presented as mean (± standard deviation [SD]); prevalence data were presented as raw numbers (percentage). Continuous variable comparisons were made using the Student’s *t* test or the Mann-Whitney test, as appropriate. ANOVA or Kruskal-Wallis tests were used for multiple comparisons of continuous variables as appropriate. The Shapiro-Wilk test was applied to test the normality of the data (*p* > 0.10). Categorical data were compared using the χ^2^ or the Fisher’s exact test as appropriate. The Bonferroni correction for multiple comparisons was applied when indicated.

The OS and DFS estimates were calculated by using the Kaplan-Meier method. The correlation between TRG and survival was evaluated by comparing the survival curves using the log-rank test or the Gehan-Breslow-Wilcoxon test, as appropriate. Moreover, the correlation of the TRG and other relevant clinicopathologic variables (pathological lymph node status; age; sex; ASA score) with the OS and the DFS after NACT or NACRT were quantified by using a Cox proportional hazards multivariable regression model.

Finally, univariate and multivariable logistic regression analyses were used to assess relevant predictors of the OS in the whole population. The following variables were assessed: TRG; pathological lymph node status; type of preoperative treatment.

The threshold for statistical significance was set to *p* < 0.05. Statistical analysis was performed by using GraphPad Prism version 9.2.0 (GraphPad software, San Diego, CA) and JMP version 14 (JMP^®^ software, SAS Institute, Cary, NC).

## RESULTS

### Demographic and Clinical Data

Overall, 563 patients were recruited during the study period; 278 received NACT, whereas 285 were treated with NACRT. The NACT regimens were mostly FLOT (77.7%), and NACRT regimens were mostly CROSS (54.7%). More details on preoperative regimens are reported in the Supplemental Digital Content (SDC) (Table 1). The mean radiation dose for the NACRT group was 45.5 (± 4) Gy.

**Table 1:** Demographic and clinical characteristics of the studied population

	NACT (N = 278)	NACRT (N = 285)	*p*
*Sex (N, %)*			**0.02**
Male	238 (86.8)	261 (91.2)	
Female	40 (13.19)	24 (8.76)	
Age (years, IQR)	60 (54-70)	63 (56-70)	0.1
ASA score (N, %)			0.91
1	3 (1.1)	3 (1.1)	
2	152 (54.7)	164 (57.5)	
3	118 (42.4)	114 (40)	
4	5 (1.8)	4 (1.4)	
Charlson Comorbidity Index (median, IQR)	4 (3-5)	4 (3-5)	0.6
Tumor location (N, %)			**0.003**
Mid thoracic	4 (1.4)	17 (6)	
Low thoracic	108 (38.8)	127 (44.6)	
EGJ	166 (59.7)	141 (49.5)	
Stage cTNM-8^AJCC (N, %)			**0.003**
2	22 (7.9)	48 (16.8)	
3	195 (70.1)	170 (59.6)	
4	61 (21.9)	67 (23.5)	

Bold values indicate significant *p* values

*NACT* neoadjuvant chemotherapy; *NACRT* neoadjuvant chemoradiotherapy

In the NACT group, 56% of patients completed the postoperative schedule of the perioperative regimens. Adjuvant treatment was performed in 15% of the patients in the NACRT group.

Demographic and clinical characteristics are reported in Table 1. The proportion of female patients was higher in the NACT group (*p* = 0.02), although the patients were mostly male in both groups. Patients receiving NACRT had more frequently a mid- and lower-thoracic tumor (*p* = 0.003) and an earlier stage of the disease at presentation (*p* = 0.003).

Short-term postoperative outcomes are reported in Table 2. Clavien-Dindo grade 3 and 4 complications occurred more frequently after NACT (*p* = 0.01), while the incidence of cardiological complications was higher after NACRT (*p* = 0.01). Globally, postoperative mortality was comparable in the two groups (*p* > 0.99); the incidence (*p* = 0.1) and severity (*p* = 0.06) of postoperative leaks were also comparable.

**Table 2:** Short-term outcomes of the studied population

	NACT (*N* = 278)	NACRT (*N* = 285)	*p*
Complications (N, %)			0.06
No	170 (61.1)	165 (57.8)	
Yes	108 (38.8)	120 (42.1)	
Complications: Clavien-Dindo			
(N, %)			**0.01**
1	10 (3.6)	19 (6.7)	
2	30 (10.8)	53 (18.6)	
3	51 (18.3)	32 (11.2)	
4	13 (4.7)	11 (3.9)	
5	4 (1.4)	5 (1.7)	
Leakage (N, %)	24 (8.6)	15 (5.3)	0.11
Leakage grade (N, %)			0.28
1	7 (2.4)	8 (2.8)	
2	13 (4.7)	6 (2.1)	
3	4 (1.4)	1 (0.3)	
Chyle leak (N, %)	8 (2.9)	5 (1.7)	0.37
Complications: type (N, %)			
Pulmonary	50 (19)	55 (19.3)	0.69
Cardiac	14 (5)	25 (8.8)	0.08
Thromboembolic	7 (2.5)	1 (0.3)	**0.03**
Infective complications	22 (7.9)	19 (6.7)	0.81
Hospital stay (days, median IQR)	12 (10–18)	13 (11–19)	0.75
ICU stay (days, median IQR)	3 (1–8)	2 (1–9)	0.44

Bold values indicate significant *p* values

*NACT* neoadjuvant chemotherapy; *NACRT* neoadjuvant chemoradiotherapy

The proportion of patients who showed pCR was significantly higher after NACRT (24.9% vs. 11.1%, *p* < 0.0001). Overall, NACRT was associated with a lower TRG both in pN0 (*p* = 0.0007) and in pN+ patients (*p* < 0.0001) (SDC; Table 2). Further details on pathological findings after the two treatments are provided in the SDC (Table 3).

**Table 3:** Cox multivariable regression model: predictors of the overall survival and the disease-free survival in the NACT group and the NACRT group

	NACT group		NACRT group	
Variable	Hazard ratio (95% CI)	*p*	Hazard ratio (95% CI)	*p*
*Overall survival*				
pN0	Ref.	–	Ref.	–
pN+	3.22 (1.95–5.61)	<0.001	2.39 (1.61–3.57)	<0.001
TRG 1-2	Ref.	–	Ref.	–
TRG 3	2.84 (1.28–6.93)	0.01	1.25 (0.79–1.98)	0.33
TRG 4-5	3.60 (1.78–8.32)	0.001	1.67 (0.98–2.68)	0.06
*Disease-free survival*				
pN0	Ref.	–	Ref.	–
pN+	2.74 (1.71-4.52)	<0.001	2.35 (1.57–3.54)	<0.001
TRG 1-2	Ref.	–	Ref.	–
TRG 3	2.02 (1.08–3.91)	0.02	1.33 (0.84–2.09)	0.22
TRG 4-5	1.83 (1.04–3.37)	0.04	1.59 (0.95–2.61)	0.07

*NACT* neoadjuvant chemotherapy; *NACRT* neoadjuvant chemoradiotherapy

### Primary Outcome

**Fig. 1:**
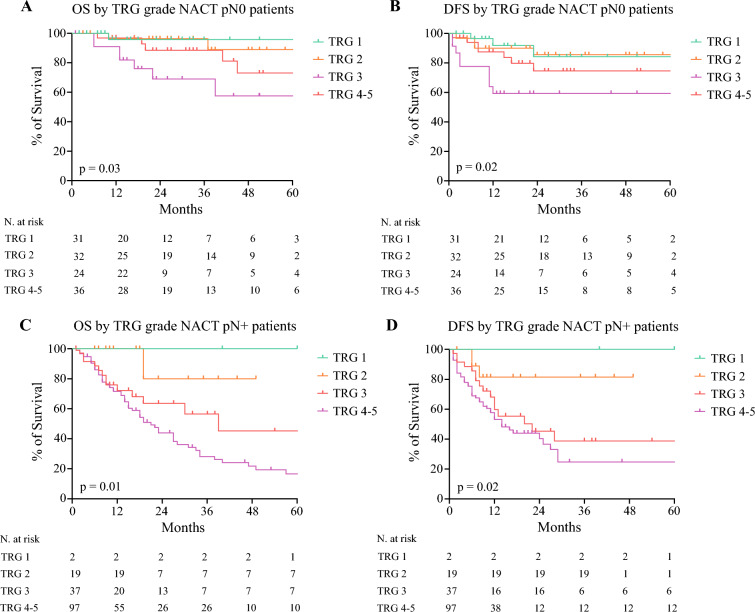
Kaplan-Meier survival curves comparing 5-year OS and 5-year DFS by TRG grade of pN0 and pN+ patients after NACT. *OS* overall survival; *DFS* disease-free survival; *NACT* neoadjuvant chemotherapy; *NACRT* neoadjuvant chemoradiotherapy

**Fig. 2:**
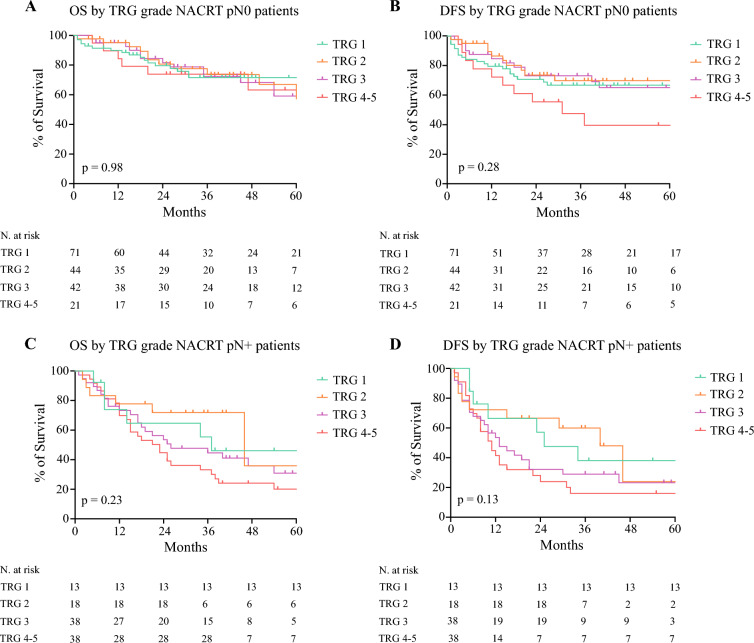
Kaplan-Meier survival curves comparing 5-year OS and 5-year DFS by TRG grade of pN0 and pN+ patients after NACRT. *OS* overall survival; *DFS* disease-free survival; *NACT* neoadjuvant chemotherapy; *NACRT* neoadjuvant chemoradiotherapy

The median follow-up time was 46 + 26 months. Overall, no differences were detected between NACT and NACRT in 5-year OS (48% vs. 52%, *p* = 0.7) and in 5-year DFS (54% vs. 49%, *p* = 0.86) (SDC; Fig. 1). In the whole population (SDC; Fig. 2), at univariate analysis, TRG was significantly correlated with the OS (*p* < 0.0001, SDC; Fig. 2A) and the DFS (*p* < 0.0001, SDC; Fig. 2B).

In the NACT group, the survival analysis of pN0 patients showed a significant correlation between TRG, OS (*p* = 0.03, Fig. 1A) and DFS (*p* = 0.02, Fig. 1B). Similarly, among pN+ patients the TRG correlated significantly with the OS (*p* = 0.01, Fig. 1C) and the DFS (*p* = 0.02, Fig. 1D). Cox regression analysis confirmed that both pN status and TRG were independent predictors of OS and DFS after NACT (Table 3).

Conversely, in the NACRT group, the OS curves of pN0 patients plotted by TRG score were overlapping (*p* = 0.98, Fig. 2A). Similarly, the DFS showed no correlation with the TRG (*p* = 0.28, Fig. 2B). Survival analysis of pN+ patients also showed no significant correlation between TRG, OS (*p* = 0.23, Fig. 2C), and DFS (*p* = 0.13, Fig. 2D). At multivariable Cox regression analysis, the pN status, but not the TRG, were independently associated with the OS and the DFS (Table 3).

**Fig. 3:**
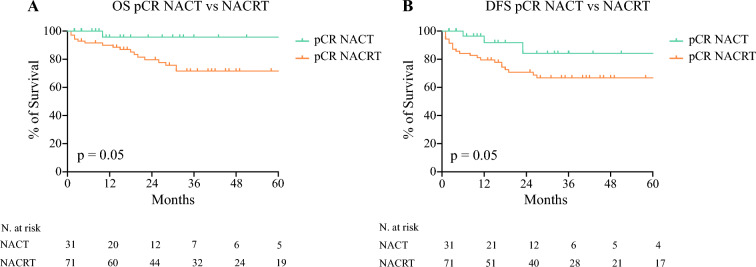
Kaplan-Meier survival curves comparing 5-year OS **A** and 5-year DFS **B** of patients with pathological complete response (pCR) after NACT and NACRT. *OS* overall survival; *DFS* disease-free survival; *NACT* neoadjuvant chemotherapy; *NACRT* neoadjuvant chemoradiotherapy

Survival analysis of patients with pCR (TRG1 N0) after treatment showed that NACRT was associated with a reduction in 5-year OS (71% vs. 95%, *p* = 0.05; Fig. 3A) and 5-year DFS (66% vs. 84%, *p* = 0.05; Fig. 3B) compared with NACT. At multivariate analysis (SDC; Table 4), the lymph node status and TRG were independently associated with the 5-year OS of the entire population (*p* < 0.01).

**Table 4:** Incidence and pattern of recurrence in the two treatment groups

	NACT (*N* = 269)*	NACRT (*N* = 280)*	*p*
*Recurrence*			0.09
No	171 (63.6)	157 (56)	
Yes	98 (36.4)	123 (44)	
*Recurrence—location*			**0.0010**
Locoregional	32 (11.9)	15 (5.4)	
Distant	54 (20.1)	85 (30.4)	
Both	12 (4.5)	23 (8.2)	

Bold value indicates significant *p* values

*NACT* neoadjuvant chemotherapy; *NACRT* neoadjuvant chemoradiotherapy

^*^Nine missing data in the NACT group and five missing data in the NACRT group

Table 4 summarizes the incidence and pattern of recurrence in the two treatment groups. Overall, the incidence of recurrence was higher in the NACRT group (44% vs. 36.4%). Univariate analysis suggested that NACRT was associated with a significantly higher rate of distant recurrences (*p* = 0.0010).

### Secondary Outcome

Survival analysis of TRG 1 pN0 patients (pCR) and TRG 2 pN0 patients (“near complete” response) showed a comparable 5-year OS (76% vs. 74%, *p* = 0.56) and DFS (71% vs. 76%, *p* = 0.36) in the whole population (SDC; Fig. 2).The same trend among complete and “near-complete” responders was detected after NACT (5-year OS 95.7% vs. 88.9%, *p* = 0.78; 5-year DFS 84.1% vs. 85.6%, *p* = 0.89) and after NACRT (5-year OS 71.5% vs. 66.9%, *p* = 0.78; 5-year DFS 66.7% vs. 69.8%, *p* = 0.52) (Figs. 1 and 2).

## Discussion

In our study, NACRT for esophageal ADC was not associated with a survival benefit despite the higher rate of pCR in this treatment group (SDC; Table 2) and an earlier stage of the disease at presentation (Table 1). The TRG was shown to correlate significantly with the 5-year OS and the 5-year DFS of the whole population (SDC; Fig. 2), confirming the reliability of TRG in stratifying survival outcomes.

The TRG showed a significant association with OS and DFS after NACT (Fig. 1). However, in the NACRT group, the different subgroups of TRG did not show correlation with survival outcomes (Fig. 2). This might suggest that an improved local tumor response cannot be considered a predictor of better survival in this treatment group.

Previously, the NeoRES RCT^20^ did not show any difference in survival outcomes between neoadjuvant chemotherapy and chemoradiotherapy despite a significantly higher rate of pCR associated with chemoradiotherapy in both ADC (22% vs. 7%, *p* < 0.001) and squamous cell carcinoma (42% vs. 16%, *p* = 0.04).

More recently, the Neo-AEGIS trial compared trimodal treatment with the CROSS regimen with perioperative chemotherapy (predominantly with the modified MAGIC regimen) for esophageal and EGJ adenocarcinoma.^4^ The study did not show any difference in 3-year survival, despite the pCR rate (16% vs. 5%, *p* = 0.012) and the major pathological response rate (41.7% vs. 12.1%, *p* < 0.0001) favoring the trimodal approach.

Following the findings of the NeoRES trial and the comparable results obtained from retrospective cohort studies,^5–9,21^ some authors postulated that the prognostic significance of the pathological tumor response may differ according to the preoperative regimen.

In a recently published retrospective multicentric cohort study, Cools-Lartigue et al.^11^ compared the survival outcomes of 132 patients with pCR after NACT (mainly MAGIC, OEO2, or FLOT) with those of 333 patients with pCR after NACRT (CROSS regimen). The authors found no significant differences in OS at 5 years (78.8% vs. 65.5%, *p* = 0.099). However, after NACT, patients had significantly improved 5-year recurrence-free survival compared with the NACRT group (87.1% vs. 75.3%, *p* = 0.026).

Finally, in the recently published ESOPEC trial,^3^ the survival outcomes of patients with resectable esophageal adenocarcinoma treated with either perioperative chemotherapy using the FLOT regimen or preoperative chemoradiotherapy were directly compared. The results demonstrated that patients in the FLOT group experienced significantly better overall survival (57.4% vs. 50.7%) and progression-free survival (51.6% vs. 35%) at 3 years.

Our results are in line with these findings. In the NACT group, we were able to identify a correlation between a lower TRG and better OS and DFS (Fig. 1). On the contrary, the survival curves of patients with TRG 1, 2, 3, and 4–5 after NACRT were somewhat comparable (Fig. 2). This supports the hypothesis that an improved local disease control after NACRT did not correspond to an actual long-term survival benefit, while this was achieved after NACT. As a consequence, patients with pCR after NACT showed better survival compared with those with pCR after NACRT (Fig. 3).

Moreover, in our cohort patients receiving NACRT showed comparable 5-year OS and DFS and a higher rate of distant recurrences compared with the NACT group, despite showing an earlier disease stage at presentation (Table 1).

One possible explanation could be the higher distant recurrence rate observed in our cohort in the NACRT group (30.4% vs. 20.1%, *p* = 0.0011; Table 4). Similar results were obtained by Cools-Lartigue et al.,^11^ who also reported more distant recurrences after CROSS (22.5% vs. 8.3%, *p* < 0.001). These findings are consistent with the recently published long-term results of the CROSS trial,^22^ which show a relatively higher cumulative incidence of distant recurrences (28%) compared with locoregional relapses (18%) at 10 years of follow-up.

Two main insights can be taken from this study and the reported literature. The first is that the chemotherapy incorporated into chemoradiotherapy regimens has a good radiosensitizing effect, although it probably lacks effective systemic control of the disease.^12^

Currently, RCTs investigating the role of preoperative induction chemotherapy associated with preoperative chemoradiotherapy for esophageal cancer, such as the RACE trial, are ongoing.^23^ The results of such studies are expected and will probably clarify the role of this treatment strategy in overcoming the limitations of conventional preoperative chemoradiotherapy.

The second is that probably the tumor regression grade, provided for instance by the Mandard TRG system, cannot be considered as a prognostic factor per se, but it must be correlated with the treatment performed, because chemo- and chemoradiotherapy could induce different degrees of cytotoxic and fibrotic effects on neoplastic tissue.^13^

The secondary endpoint of our study was to evaluate whether the survival outcomes of patients showing a complete and a “near-complete” response to treatment (namely TRG 1 and 2 pN0 patients) were comparable. The main implication is the feasibility of incorporating the two main tiers of the pathological tumor response into a single tier of “main pathological response.”

Our results supported this hypothesis; indeed, patients with TRG 1 and TRG 2 showed almost overlapping 5-year OS and 5-year DFS curves in the whole population. This finding was confirmed after segregating for the type of preoperative treatment, thus showing that the complete and nearly complete responders had comparable survival outcomes after both NACT and NACRT.

Previously published data supported our results. In a recently published single-center retrospective cohort study, Sihag et al. analyzed the OS of 788 patients with esophageal ADC after trimodal treatment by their tumor response (TR), measured according to the College of American Pathologists TRG.^24,25^ The 5-year OS was markedly similar between patients with 90% to 99% TR and those with 100% TR, especially when the ypN status was negative. The authors concluded that the distinction between a complete and a near-complete response did not carry prognostic weight. The main implication is that the use of the “major pathologic response” instead of only the “complete response rate” could potentially expedite the conduct of clinical trials by substantially increasing the prevalence of the primary endpoint, thus potentially minimizing type II error. We believe that our results bring a major caveat to this conclusion, because we have shown that TRG is not reliably correlated with survival after NACRT in node negative patients. Therefore, the use of TR as a surrogate outcome for survival in this setting should be further investigated.

Another point of discussion concerns the introduction of adjuvant immunotherapy for patients with residual tumor in the surgical specimen after NACRT. After the publication of the CheckMate 577 study, this treatment has been widely adopted; however, the occurrence of potentially severe side effects associated with immunotherapy should be considered when referring patients to such a regimen.^26–28^ In this context, our study poses the question of the actual survival benefit of adjuvant immunotherapy for patients with TRG 2 N0, given that their survival is comparable to that of complete pathological responders. Further studies should investigate the actual prognostic benefit of adjuvant immunotherapy in this subset.

There are limitations of this study that should be considered, including its retrospective design and multi-institutional participation, with potential variability in surgical techniques, postoperative management, and pathologic evaluation of samples. The treatment regimens included in the NACT/NACRT groups, and the radiation doses were heterogeneous, reflecting the real-life practice adopted at the time of recruitment. However, FLOT and CROSS represented the significant majority of the included schemes. Furthermore, we believe that the strict inclusion criteria adopted in the study might have overcome this limitation. Finally, OS was defined regardless the cause of death, potentially overestimating cancer-related mortality. However, noncancer-related deaths are expected to be comparable among the treatment groups, because no differences regarding ASA and CCI were detected.

## Conclusions

The present multicentric cohort study demonstrated the correlation between tumor response and survival in patients treated with NACT. The association between TRG and OS/DFS was not confirmed after NACRT and the incidence of distant recurrences was higher in this treatment group. These findings might support the hypothesis that an improved local tumor-response after NACRT may not correspond to an improved systemic disease control, while this is achieved after NACT. Future investigations should be aimed at evaluating the correlation between tumor response and survival after NACT and NACRT and exploring the potential benefit of combining induction chemotherapy and chemoradiotherapy.

The survival outcomes of TRG1 and 2 was similar after both treatments, potentially indicating the feasibility of including these patients into a single tier of “main pathological response.” However, this finding requires further investigation, especially after NACRT, as we demonstrated that there is no correlation between tumor response and survival in this group.

## Supplementary Information

Below is the link to the electronic supplementary material.

Supplementary file 1 (DOCX 260 kb)
