# Supplementary material for: Prognostic Value of Tumor Regression Grade After Chemotherapy Versus Chemoradiotherapy in Patients Undergoing Neoadjuvant Treatment for Locally Advanced Esophageal Adenocarcinoma
Source: Ann Surg Oncol. 2025 May 5;32(8):5909–18. doi: 10.1245/s10434-025-17264-2 (PMC12222383; doi:10.1245/s10434-025-17264-2)
Supplement: Supplementary file 1 — (DOCX 260 kb) [file 10434_2025_17264_MOESM1_ESM.docx]

**Supplemental digital content (SDC)**

**Table 1.** Preoperative regimens in the NACT and the NACRT group.

| Treatment Group | Regimen | N. of patients | % |
| --- | --- | --- | --- |
| NACT | Fluoruracil, leucovorin, oxaliplatin, docetaxel | 216 | 77.7 |
|  | Folinic acid, fluoruracil, oxaliplatin | 18 | 6.5 |
|  | Cisplatin, fluoruracil  OR  Cisplatin, capecitabine | 17 | 6.1 |
|  | Epirubicin, cisplatin, fluoruracil  OR  Epirubicin, cisplatin, capecitabine | 13 | 4.7 |
|  | Docetaxel, cisplatin, fluoruracil | 12 | 4.3 |
|  | Epirubicin, capecitabine, oxaliplatin | 2 | 0.7 |
|  |  |  |  |
| NACRT | Carboplatin, paclitaxel | 156 | 54.7 |
|  | Docetaxel, cisplatin, fluoruracil | 68 | 23.9 |
|  | Cisplatin, fluoruracil | 43 | 15.1 |
|  | Folinic acid, fluoruracil, oxaliplatin | 10 | 3.5 |
|  | Epirubicin, capecitabine, oxaliplatin | 8 | 2.8 |

NACT: neoadjuvant chemotherapy; NACRT: neoadjuvant chemoradiotherapy.

**Table 2.** Incidence of node metastases by TRG grade.

|  | **NACT (N=278)** | | **NACRT (N=285)** | | **All (N=563)** | |
| --- | --- | --- | --- | --- | --- | --- |
|  | **N0** | **N+** | **N0** | **N+** | **N0** | **N+** |
| **TRG 1** | 31 (11.1) | 2 (0.7) | 71 (24.9) | 13 (4.6) | 102 (18.1) | 15 (2.7) |
| **TRG 2** | 32 (11.5) | 19 (6.8) | 44 (15.4) | 18 (6.3) | 76 (13.5) | 37 (6.6) |
| **TRG 3** | 24 (8.6) | 37 (13.3) | 42 (14.7) | 38 (13.3) | 66 (11.7) | 75 (13.3) |
| **TRG 4 o 5** | 36 (12.9) | 97 (34.9) | 21 (7.4) | 38 (13.3) | 57 (10.1) | 135 (23.9) |
|  | **p < 0.0001** | | **p < 0.0001** | | **p < 0.0001** | |

NACT: neoadjuvant chemotherapy. NACRT: neoadjuvant chemoradiotherapy.

**Table 3.** Details of the pathological findings on the specimens.

|  | **NACT**  **(n= 278)** | **NACRT**  **(n= 285)** | **p** |
| --- | --- | --- | --- |
| **ypT (8^ed AJCC)**  0  1  2  3  4 | 33 (11.9)  36 (12.9)  49 (17.6)  145 (52.2)  15 (5.4) | 84 (29.5)  34 (11.9)  61 (21.4)  104 (36.5)  2 (0.7) | **< 0.0001** |
| **ypN (8^ed AJCC)**  0  1  2  3 | 123 (44.2)  58 (20.9)  46 (16.5)  51 (18.3) | 178 (62.5)  61 (21.4)  29 (10.2)  17 (6) | **< 0.0001** |
| **ypTNM (8^ed AJCC)**  1  2  3  4 | 84 (30.2)  36 (12.9)  102 (36.7)  56 (20.1) | 131 (46)  46 (16.1)  90 (31.6)  18 (6.3) | **< 0.0001** |
| **Perineural invasion** | 117 (42.1) | 69 (24.2) | **< 0.0001** |
| **Lymphovascular invasion** | 120 (43.2) | 72 (25.3) | **< 0.0001** |
| **TRG (Mandard)**  1  2  3  4 o 5 | 33 (11.9)  51 (18.3)  61 (21.9)  133 (47.8) | 84 (29.5)  62 (21.8)  80 (28.1)  59 (20.7) | **< 0.0001** |
| **pCR** | 31 (11.1) | 71 (24.9) | **< 0.0001** |

NACT: neoadjuvant chemotherapy; NACRT: neoadjuvant chemoradiotherapy;

pCR: pathological complete response.

**Table 4.** Univariate and multivariate analysis of factors associated with 5y OS.

|  | **Univariate analysis** | | **Multivariate analysis** | |
| --- | --- | --- | --- | --- |
| **Variable** | Odds Ratio (95%CI) | p | Odds Ratio (95%CI) | p |
| pN0 (ref) vs pN+ | 3.87 (2.70-5.54) | <0.01 | 2.91 (1.98-4.27) | <0.01 |
| TRG 1-2 (ref) vs TRG 3-5 | 3.40 (2.33-4.96) | <0.01 | 2.31 (1.54-3.47) | <0.01 |
| NACRT (ref) vs NACT | 1.26 (0.89-1.76) | 0.19 |  |  |

NACT: neoadjuvant chemotherapy; NACRT: neoadjuvant chemoradiotherapy;

CI: confidence interval; ref: reference


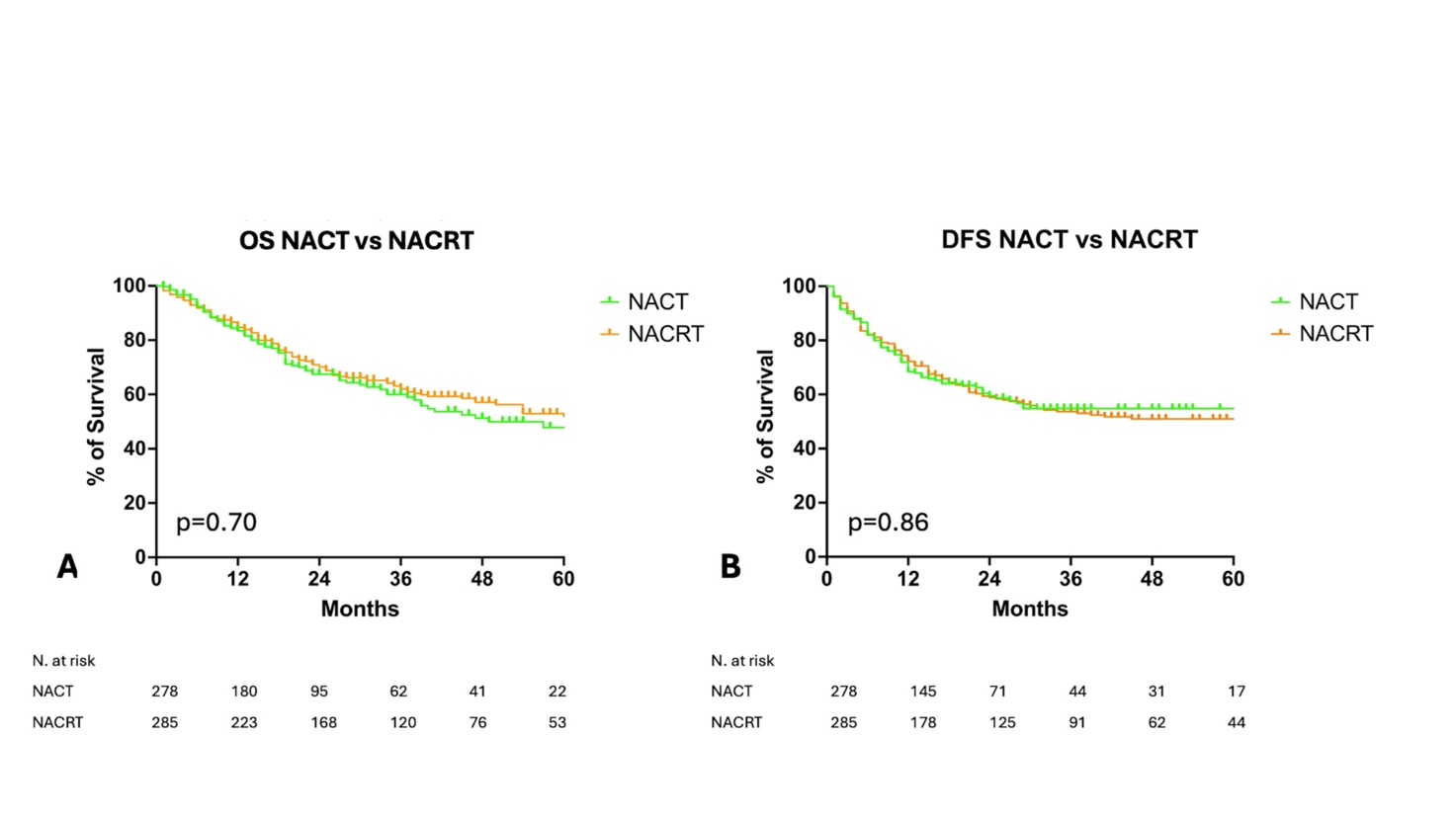


**Figure 1.** Kaplan-Meier survival curve comparing 5y OS (A) and 5y DFS (B) of patients after NACT and NACRT.

OS: overall survival; DFS: disease-free survival; NACT: neoadjuvant chemotherapy; NACRT: neoadjuvant chemoradiotherapy;

**
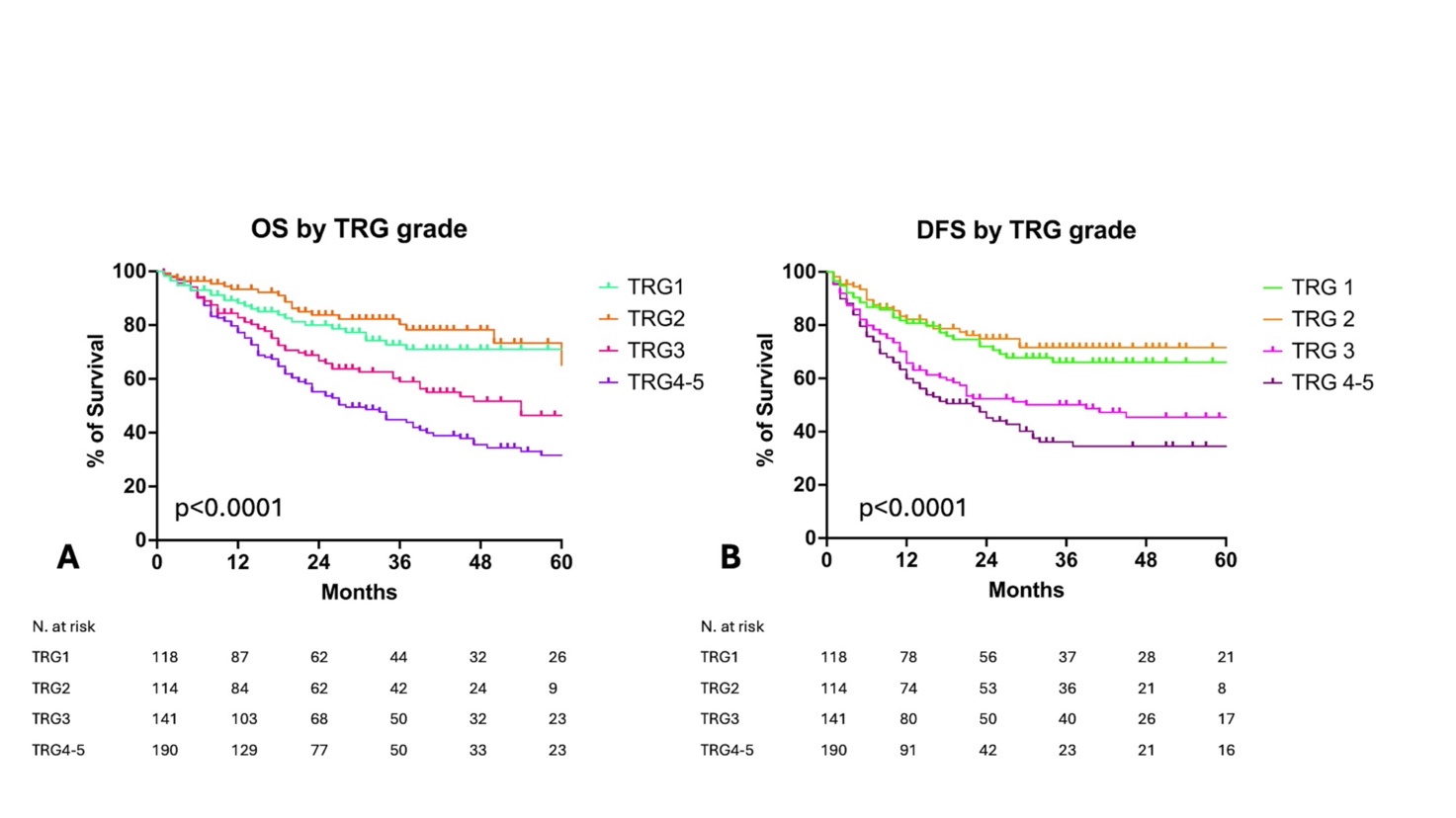
**

**Figure 2.** Kaplan-Meier survival curves comparing 5y OS (A) and 5y DFS (B) by TRG grade of the whole population.

OS: overall survival; DFS: disease-free survival; NACT: neoadjuvant chemotherapy; NACRT: neoadjuvant chemoradiotherapy;
